# Supplementary material for: All-optical reporting of inhibitory receptor driving force in the nervous system
Source: Nat Commun. 2024 Oct 16;15:8913. doi: 10.1038/s41467-024-53074-y (PMC11484818; doi:10.1038/s41467-024-53074-y)
Supplement: Supplementary file 1 — Supplementary information [file 41467_2024_53074_MOESM1_ESM.pdf]

Supplementary information

All-optical reporting of inhibitory receptor driving force in the nervous system

Joshua S. Selfe[1,2], Teresa J. S. Steyn[1,2], Eran F. Shorer[1,2,3], Richard J. Burman[4], Kira M. Düsterwald[1,2,5], Ariel Z. Kraitzick[1,2], Ahmed S. Abdelfattah[6,7], Eric R. Schreiter[8], Sarah E. Newey[4], Colin J. Akerman[4], Joseph V. Raimondo[1,2,9,*]

[1]Division of Cell Biology, Department of Human Biology, University of Cape Town, South Africa
[2]Neuroscience Institute, University of Cape Town, South Africa
[3]Department of Neurology, School of Medicine, Johns Hopkins Hospital, Baltimore, Maryland, United States of America
[4]Department of Pharmacology, University of Oxford, United Kingdom
[5]Gatsby Computational Neuroscience Unit, University College London, United Kingdom
[6]Department of Neuroscience, Brown University, Providence, Rhode Island, United States of America
[7]Carney Institute for Brain Science, Brown University, Providence, Rhode Island, United States of America
[8]Janelia Research Campus, Howard Hughes Medical Institute, Ashburn, Virginia, United States of America
[9]Wellcome Centre for Infectious Disease Research in Africa, Institute of Infectious Disease and Molecular Medicine, University of Cape Town, Cape Town, South Africa

Corresponding author email address

joseph.raimondo@uct.ac.za

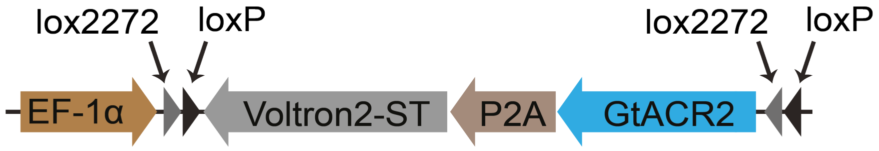

Supplementary Figure 1. *The ORCHID construct.* Voltron2-ST is expressed together with GtACR2 via a P2A linker sequence, with a double-floxed inverted-orientation design and expression under the EF-1α promoter.

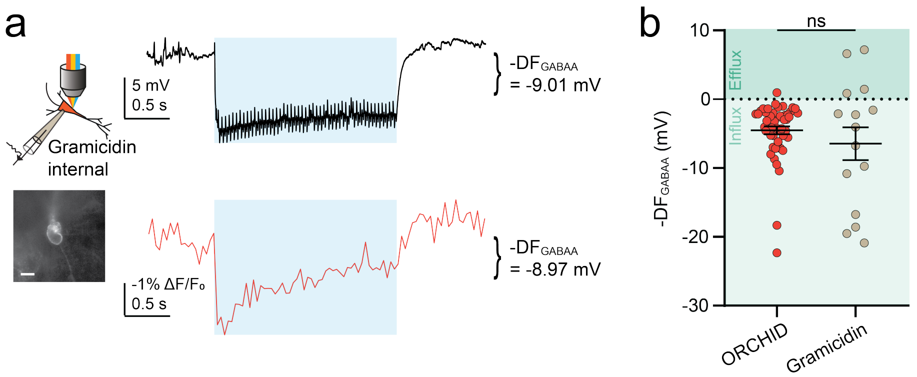

Supplementary Figure 2. *ORCHID estimates of DF_{GABAA} are consistent with those acquired using gramicidin-perforated patch-clamp recordings*. **a**, Gramicidin perforated patch-clamp recordings were made from ORCHID-expressing neurons in hippocampal organotypic slices. In current-clamp configuration, DF_{GABAA} was measured through the perforated patch-clamp recording (top panel, black trace) whilst concurrently, and optically, DF_{GABAA} was measured using ORCHID (bottom panel, orange trace). These measurements were found to be consistent with each other. Patched neuron shown inset; scale bar: 10 µm. **b**, Population DF_{GABAA} measurements made from hippocampal organotypic-slice CaMKIIα+ pyramidal neurons using ORCHID were compared to separate DF_{GABAA} measurements made from hippocampal organotypic-slice pyramidal neurons using gramicidin-perforated patch-clamp recordings. There was no statistical difference between these two populations (ORCHID: -4.52 ± 0.57 mV vs gramicidin: -6.47 ± 2.39, Mann Whitney test, two-tailed, P = 0.7969, n = 51 and 15 respectively). Green shading indicates direction of anion flux. $-DF_{GABAA}$ values reported. *ns* = not significant ($P > 0.05$); error bars indicate mean ± SEM.

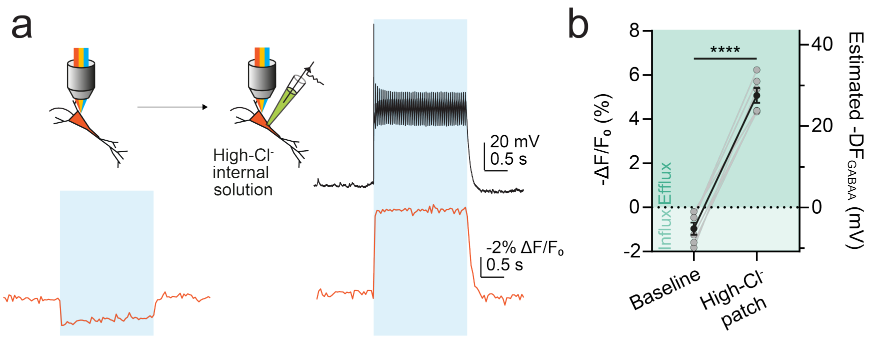

Supplementary Figure 3: *ORCHID can detect changes in DF_{GABAA} induced by an increase in $[Cl^-]_i$ imposed by dialysis.* **a**, Schematic (top left) showing ORCHID used to record DF_{GABAA} in a neuron in a hippocampal organotypic slice at baseline (bottom left, orange trace) and again after being patched with high-Cl^- (141 mM) intracellular solution (right). Both voltage imaging (bottom right panel, orange trace) and current-clamp recordings (top right panel, black trace) showed a transition to a strongly depolarizing DF_{GABAA}. Action potentials are truncated due to the low-pass behavior of the image acquisition frequency (25 Hz) and averaging of responses (see Methods). **b**, A highly significant difference between DF_{GABAA} at baseline and after the high-Cl^- patch was found (paired t-test, two-tailed, P = 0.000016, n = 6). ****$P \leq 0.0001$; error bars indicate mean ± SEM.

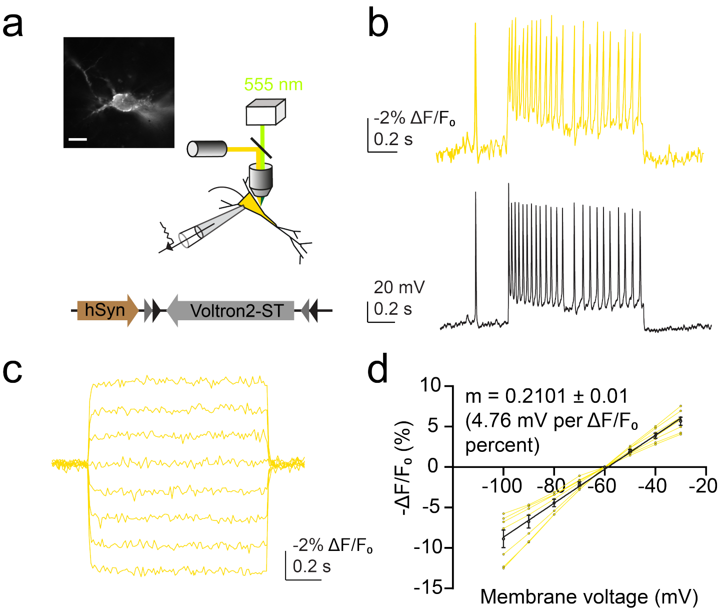

Supplementary Figure 4: *Characterizing Voltron2$_{549}$-ST for measurement of V_m.* **a**, Schematic of experimental setup for patch-clamp characterization of Voltron2$_{549}$-ST in mouse hippocampal organotypic slices. Inset: widefield fluorescence image of the neuron patched in (b); scale bar: 10 µm. **b**, Current-clamp recording (bottom panel, black trace) and the Voltron2$_{549}$-ST fluorescence response (top panel, yellow trace) following a 200 pA current injection. **c**, Voltron2$_{549}$-ST fluorescence response to 10 mV voltage steps (V_{hold} = -60 mV). **d**, Fluorescence-voltage relationship of Voltron2$_{549}$-ST. Linear regression results in a slope of 0.2101 ± 0.01 mV, which equates to 4.76 mV

per $\Delta F/F_0$ percent (R^2 = 0.9236, *n* = 7 cells). Yellow traces are recordings from individual neurons; the black trace is the linear regression of the data, with error bars showing SEM.

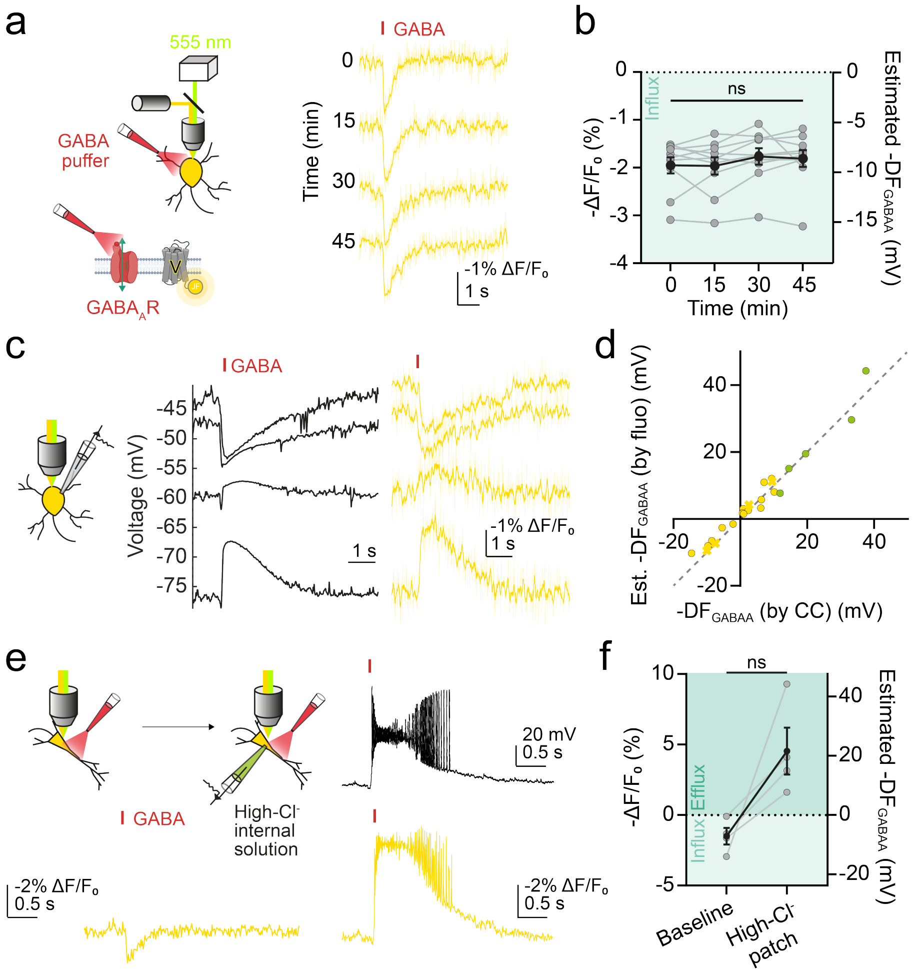

Supplementary Figure 5: *Characterizing Voltron2$_{549}$-ST for measurement of DF_{GABAA} via activation of endogenous $GABA_A$Rs.* **a**, Left, schematic demonstrating imaging of Voltron2$_{549}$-ST together with activation of endogenously expressed $GABA_A$Rs through picolitre delivery of 500 µM GABA (red) directed at the cell soma, with 5 µM CGP-55845 used to block $GABA_B$Rs. Right, repeated fluorescence transients (yellow traces) from neurons in hippocampal organotypic slices in response to GABA application (red bar) recorded over 45 min. **b**, Population data showing estimates of DF_{GABAA} calculated from $\Delta F/F_0$ remained stable (Friedman test, *P* = 0.2933, *n* = 10 cells). Dunn's multiple comparison test was used post-hoc and found no statistical difference between any pair of means (Supplementary Table 2). **c**, Voltron2$_{549}$-ST and activation of $GABA_A$Rs were used to estimate DF_{GABAA} in neurons current-clamped at a range of V_m values (left panel, V_m; right panel, fluorescence). **d**, The fluorescence-voltage relationship of Voltron2$_{549}$-ST (Supplementary Fig. 4d) was used to convert $\Delta F/F_0$ measurements at each V_m to estimated (est.) DF_{GABAA} values (by fluorescence, i.e., fluo). This was done for neurons patched with low-Cl^- internal solution (yellow; crosses denote the points displayed in plot [c]; *n* = 18 recordings from 6 cells), and neurons patched with high-Cl^- internal solution (green; *n* = 5 recordings from 5 cells). Where available for low-Cl^- internal recordings, holding potentials below, above, and close to E_{GABAA} are plotted for each cell. These optically estimated DF_{GABAA} values did not differ from DF_{GABAA} recorded using current-clamp (CC) recordings (R^2 = 0.9624; Runs test,

deviation from linearity: $P = 0.6650$). Dashed-grey identity line. **e**, Schematic (top left panel) showing Voltron2$_{549}$-ST and activation of GABA$_A$Rs used to estimate DF$_{GABAA}$ in neurons at baseline (bottom left panel, yellow trace) and again after being patched with high-Cl$^-$ (141 mM) internal solution. Both voltage imaging (bottom right panel, yellow trace) and current-clamp recordings (top right panel, black trace) showed a transition to a strongly depolarizing response to GABA$_A$R activation after Cl$^-$ loading. **f**, Although there was a large depolarization in DF$_{GABAA}$ upon filling with high-Cl$^-$ internal solution in all neurons patched this was not statistically significant due to a small sample size (paired t-test, two-tailed, $P = 0.0627$, $n = 4$). ns = not significant ($P > 0.05$); error bars indicate mean ± SEM.

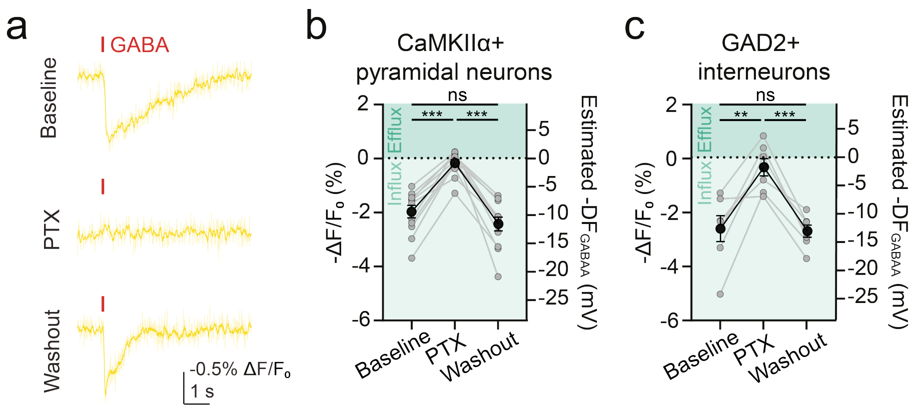

Supplementary Figure 6: *Voltron2$_{549}$-ST estimates of agonist-evoked DF$_{GABAA}$ reflect GABA$_A$R activity.* **a**, Voltron2$_{549}$-ST and GABA puffs were used to record DF$_{GABAA}$ in hippocampal organotypic-slice neurons at baseline, during picrotoxin (PTX; 100 µM) wash-in, and again after PTX washout. **b**, Population data showing recordings of DF$_{GABAA}$ in CaMKIIα+ pyramidal neurons measured hyperpolarizing DF$_{GABAA}$ at baseline, with this response disappearing upon PTX wash-in (Wilcoxon matched-pairs signed rank test, two-tailed, $P = 0.0005$, $n = 12$ cells). The hyperpolarizing DF$_{GABAA}$ response returned upon PTX washout (baseline vs washout: paired t-test, two-tailed, $P = 0.1256$; PTX vs washout: Wilcoxon matched-pairs signed rank test, two-tailed, $P = 0.0005$). **c**, Population data for GAD2+ interneurons (baseline vs PTX: paired t-test, two-tailed, $P = 0.0032$, $n = 7$ cells; baseline vs washout: paired t-test, two-tailed, $P = 0.8604$; PTX vs washout: paired t-test, two-tailed, $P = 0.0002$). ns = not significant ($P > 0.05$); $^{**}P \leq 0.01$; $^{***}P \leq 0.001$; error bars indicate mean ± SEM.

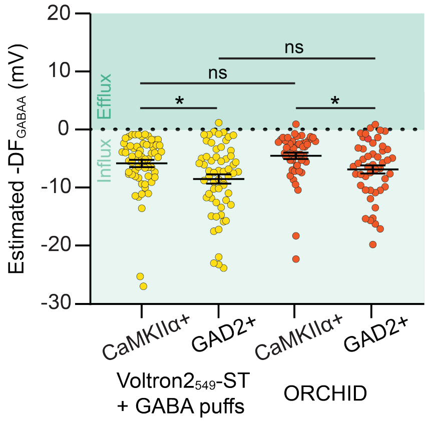

Supplementary Figure 7: *DF$_{GABAA}$ recorded using Voltron2$_{549}$-ST and GABA puffs is equivalent to DF$_{GABAA}$ recorded using ORCHID.* DF$_{GABAA}$ measurements made using Voltron2$_{549}$-ST and GABA puffs were equivalent to DF$_{GABAA}$ measurements made using ORCHID for both CaMKIIα+ pyramidal neurons (Mann Whitney test, two-tailed, $P = 0.0660$, $n = 62$ and 51 respectively) and GAD2+ interneurons (Mann Whitney test, two-tailed, $P = 0.1819$, $n = 60$ and 49 respectively). This data is shown in Fig. 2c,f, as are the cell-type comparison statistics (Voltron2$_{549}$-ST and GABA puffs, CaMKIIα+ vs GAD2+: Mann-Whitney test, two-tailed, $P = 0.0068$, $n = 62$ and 60 cells respectively. ORCHID, CaMKIIα+ vs GAD2+: Mann-Whitney test, two-tailed, $P = 0.0057$, $n = 51$ and 49 cells respectively). ns = not significant ($P > 0.05$); *$P \leq 0.05$; error bars indicate mean ± SEM.

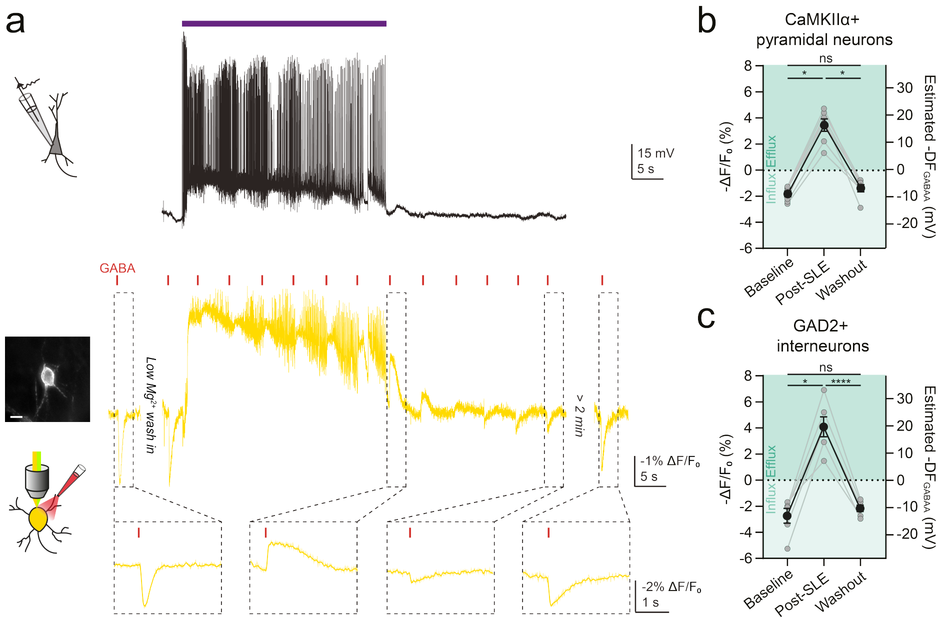

Supplementary Figure 8: *Cell-type-specific dynamics in DF$_{GABAA}$ recorded using Voltron2$_{549}$-ST and GABA$_A$R activation during SLEs.* **a**, Voltron2$_{549}$-ST and GABA$_A$R activation were used to investigate activity-dependent variation in DF$_{GABAA}$ in neurons in mouse hippocampal organotypic slices. SLEs were induced using the low-Mg^{2+} model of *in vitro* SLEs. A whole-cell current-clamp recording from a CA1/3 pyramidal neuron provided an independent readout of SLEs (top panel, black trace; purple bar indicates SLE). Concurrent Voltron2$_{549}$-ST recordings were used to simultaneously record DF$_{GABAA}$ in target cells, whilst also providing a recording of the SLE

(bottom panel, yellow traces). Central inset image: widefield fluorescence image of the GAD2+ interneuron from which the recording was made; scale bar: 10 µm. Recordings of DF_{GABAA} were made prior to low-Mg^{2+} aCSF wash-in, immediately post-SLE (< 15 s after SLE cessation), and > 2 min after SLE cessation (washout). The first, second, and fourth responses shown in the dashed boxes represent baseline, post-SLE, and washout respectively. **b**, Population data of DF_{GABAA} in CaMKIIα+ pyramidal neurons showed a shift from hyperpolarizing DF_{GABAA} at baseline to depolarizing DF_{GABAA} post-SLE (Wilcoxon matched-pairs signed rank test, two-tailed, $P = 0.0156$, $n = 7$ cells). > 2 min after the SLE (washout), DF_{GABAA} had returned to baseline levels (Wilcoxon matched-pairs signed rank tests, two-tailed; baseline vs washout: $P = 0.1563$; post-SLE vs washout: $P = 0.0156$). **c**, Similar results were recorded in GAD2+ interneurons (Wilcoxon matched-pairs signed rank tests, two-tailed; baseline vs post-SLE: $P = 0.0313$; baseline vs washout: $P = 0.2188$; post-SLE vs washout: paired t-test, two-tailed $P = 0.000099$, $n = 6$). ns = not significant ($P > 0.05$); $^*P \leq 0.05$; $^{**}P \leq 0.01$; $^{****}P \leq 0.0001$; error bars indicate mean ± SEM.

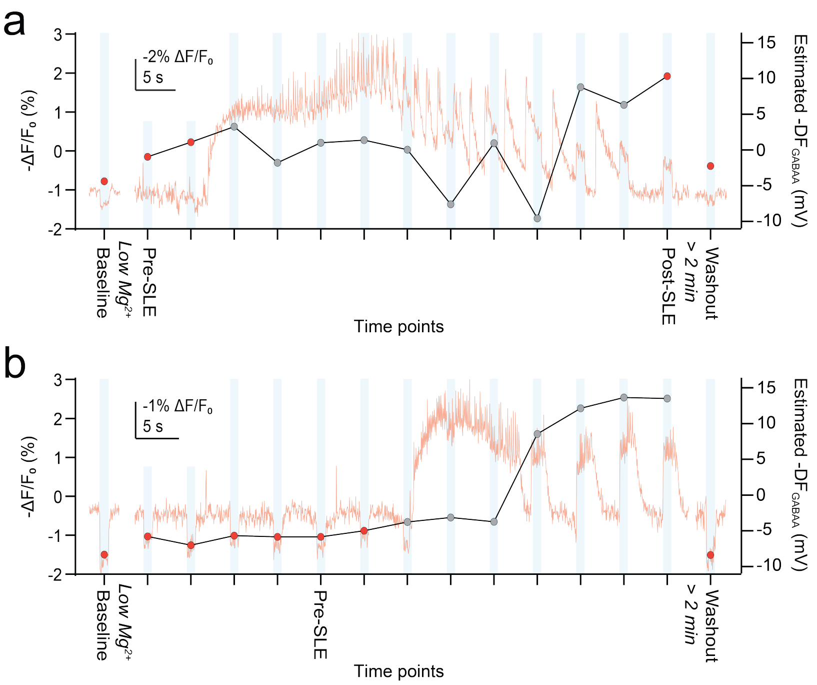

Supplementary Figure 9: *Dynamics of DF_{GABAA} in neurons before and after an SLE.* **a**, An ORCHID recording of a low-Mg^{2+} induced SLE in a CaMKIIα+ pyramidal neuron in a hippocampal organotypic slice (shown in Fig. 4a). The DF_{GABAA} values (circles) are overlayed on the voltage-imaging trace of the SLE and associated DF_{GABAA} measurements (orange trace, blue rectangles indicate blue-light activation of ORCHID). Grey circles indicate DF_{GABAA} measurements that were contaminated by SLE-associated spiking. The x-axis indicates DF_{GABAA} measurements that were used during analysis, as well as indicating when the low-Mg^{2+} wash-in occurred, and when washout occurred (> 2 min after SLE cessation). **b**, As in (a), but in a GAD2+ interneuron (shown in Fig. 4c). After the SLE, further network burst firing activity occurs upon blue-light stimulation, further contaminating DF_{GABAA} measurements (Supplementary Fig. 10).

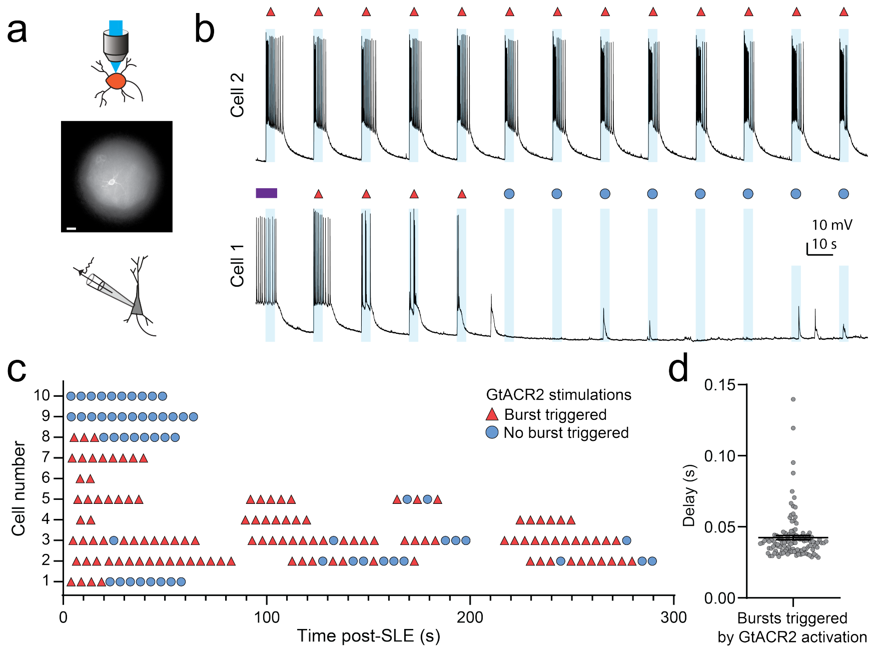

Supplementary Figure 10: *Stimulation of GtACR2 expressed in GAD2+ interneurons after an SLE causes slice-wide network burst firing.* **a**, ORCHID recordings were made from GAD2+ interneurons in the stratum radiatum of hippocampal organotypic slices during seizure-like events (SLEs). This involved stimulating GtACR2 expressed as part of ORCHID (top panel). The area of stimulation was restricted to a ~140 µm diameter region around the GAD2+ interneuron using a field stop aperture (middle panel). A whole-cell current-clamp recording from a CA1/3 pyramidal neuron provided an independent readout of SLEs (bottom panel). **b**, Separate examples (not concurrent) of current-clamp recordings (black traces) from two separate pyramidal neurons (not expressing ORCHID) where slice-wide network burst firing occurred upon distant stimulation of GtACR2 (blue rectangles) expressed in GAD2+ interneurons after an SLE (purple rectangle where present in recording). Red triangles indicate that a network burst was triggered, while blue circles indicate that no network burst was triggered (Methods). **c**, Similar to (b), a summary of the triggering of network bursts upon GtACR2 stimulation post-SLE in current-clamp recordings from 10 separate CA1/3 pyramidal neurons (not expressing ORCHID). Slice-wide network burst firing occurred in 8/12 ORCHID recordings from GAD2+ interneurons; here current-clamp recordings from 10 cells are analyzed as some ORCHID-expressing GAD2+ interneurons were recorded simultaneously in the same field of view, and thus have a single associated current-clamp recording. Gaps are time periods during which recordings were not made. A full recording consists of 13 GtACR2 stimulations; some recordings were truncated. Cell numbering is the same as in (b). **d**, Delay between blue light onset and the start of a network burst event (0.0424 ± 0.0015 s, *n* = 115 network burst events from 10 cells. Error bars indicate mean ± SEM.

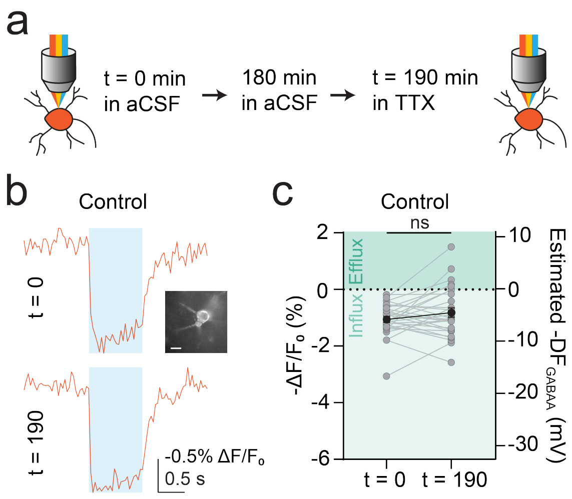

Supplementary Figure 11: *ORCHID estimates of DF_{GABAA} are stable over 190 min.* **a**, Schematic of experimental design with ORCHID used to make paired recordings of DF_{GABAA} 190 min apart in the same GAD2+ interneurons in hippocampal organotypic slices. **b**, Top, baseline recording from a GAD2+ interneuron before slice was incubated in normal aCSF for 180 min. Bottom, another recording from the same cell after the slice had been transferred to aCSF containing TTX (1 µM) for 10 min. Inset: widefield fluorescence image of recorded neuron; scale bar = 10 µm. **c**, Population data shows DF_{GABAA} was not significantly different at t = 0 and t = 190 (Wilcoxon matched-pairs signed rank test, two-tailed, P = 0.2297, n = 29 cells). ns = not significant (P > 0.05); error bars indicate mean ± SEM.

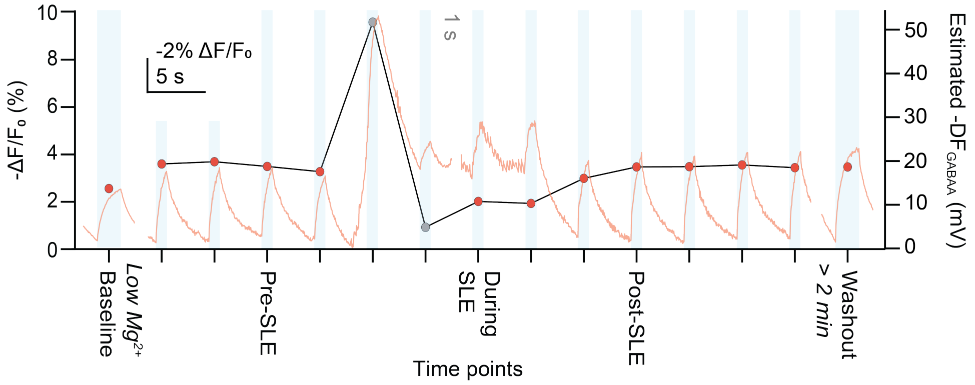

Supplementary Figure 12: *Dynamics of DF_{GABAA} in GFAP+ astrocytes before, during, and after an SLE.* **a**, An ORCHID recording of a low-Mg^{2+} induced SLE in a GFAP+ astrocyte in a hippocampal organotypic slice (shown in Fig. 5d). The DF_{GABAA} values (circles) are overlayed on the voltage-imaging trace of the SLE and associated DF_{GABAA} measurements (orange trace, blue rectangles indicate blue-light activation of ORCHID). Grey circles indicate DF_{GABAA} measurements that should be interpreted with caution due to powerful concurrent SLE-associated conductances. The x-axis indicates DF_{GABAA} measurements that were used during analysis, as well as indicating when the low-Mg^{2+} wash-in occurred, and when washout occurred (> 2 min after SLE cessation).

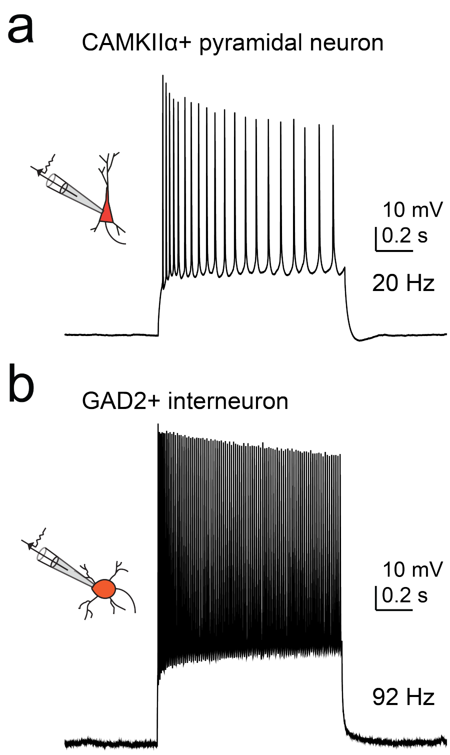

Supplementary Figure 13: *Electrophysiological characterization of a CaMKIIα+ pyramidal neuron and GAD2+ interneuron expressing ORCHID.* **a**, To confirm the cell type of CaMKIIα+ neurons expressing ORCHID in hippocampal organotypic slices, current-clamp recordings of current steps were performed in a subset of neurons. This example shows a 250 pA current step, with a resulting action potential firing rate of 20 Hz. **b**, As in (a), but with a GAD2+ neuron expressing ORCHID. Here, a 250 pA current step resulted in an action potential firing rate of 92 Hz.

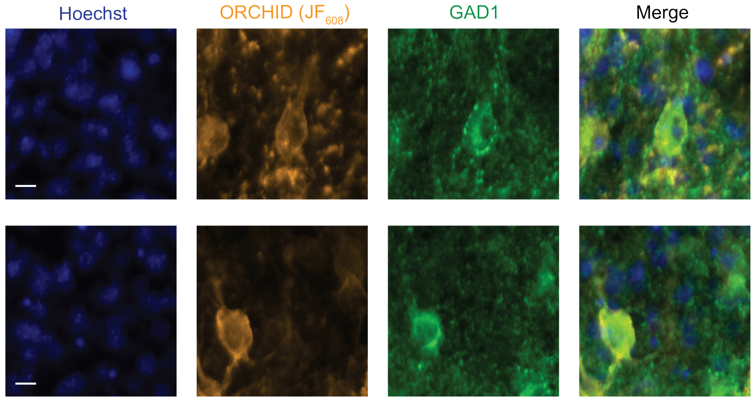

Supplementary Figure 14: *Immunohistochemistry confirmation of correctly targeted expression of ORCHID in GAD2+ interneurons.* Immunohistochemistry was performed in a subset of hippocampal organotypic slices to confirm co-expression of GAD1 and double-floxed inverted-orientation (DIO) ORCHID in hippocampal organotypic slices from GAD2-IRES-Cre mice; two examples of co-expression are shown. DAPI (far left panels), DIO-ORCHID (middle left panels), GAD1 (middle right panels), and an overlay of all three (far right panels) are shown; scale bars: 10 µm.

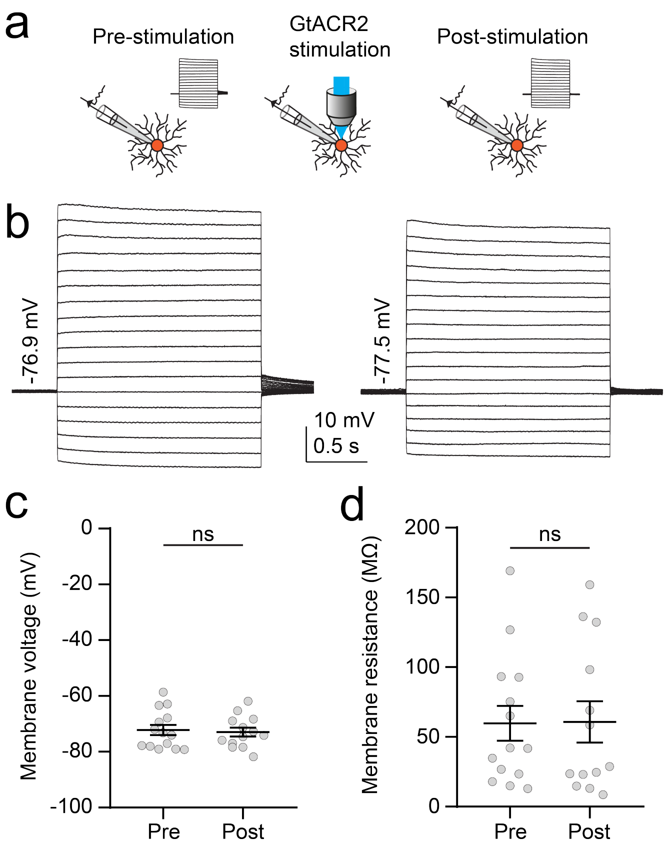

Supplementary Figure 15: *Electrophysiological characterization of ORCHID-expressing GFAP+ astrocytes and the effect of GtACR2 stimulation on their physiology*. **a**, ORCHID-expressing GFAP+ astrocytes were whole-cell current-clamped, and responses to 50 pA current steps from -250 pA to 600 pA were recorded before or after repeated GtACR2 stimulation for 3-5 min. **b**, Current step recordings revealed an inability to fire action potentials in all cells (*n* = 14/14) consistent with an astrocytic phenotype. Additionally, V_m responses of current steps were similar before (left panel) and after (right panel) GtACR2 stimulation. **c**, The GFAP+ astrocytes had a low resting V_m at baseline (-72.25 ± 1.84 mV), which was conserved post-stimulation (-72.97 ± 1.57 mV; unpaired t-test, two-tailed, *P* = 0.7701, *n* = 14 and 13 cells respectively). **d**, The GFAP+ astrocytes had a low resting R_m at baseline 59.76 ± 12.53 MΩ), which was conserved post-stimulation (-60.75 ± 14.81 MΩ; unpaired t-test, two-tailed, *P* = 0.9592, *n* = 14 and 13 cells respectively).

Supplementary Table 1: Tukey's multiple comparisons test for Figure 1I.

Tukey's multiple comparisons test	Mean difference	95% confidence interval of difference	Significant?	Summary	Adjusted *P* Value
Time = 0 vs. Time = 15	-0.03271	-0.1716 to 0.1062	No	ns	0.8986
Time = 0 vs. Time= 30	0.07126	-0.1158 to 0.2583	No	ns	0.6852
Time = 0 vs. Time= 45	0.057	-0.1063 to 0.2203	No	ns	0.7384
Time = 15 vs. Time= 30	0.104	-0.1119 to 0.3199	No	ns	0.5132
Time = 15 vs. Time= 45	0.08972	-0.09354 to 0.2730	No	ns	0.5001
Time = 30 vs. Time= 45	-0.01426	-0.07482 to 0.04631	No	ns	0.8987

Supplementary Table 2: Dunn's multiple comparisons test for Supplementary Figure 7.

Dunn's multiple comparisons test (two-tailed)	Rank sum difference	Significant?	Summary	Adjusted *P* Value
Time = 0 vs. Time = 15	1	No	ns	>0.9999
Time = 0 vs. Time = 30	-4	No	ns	>0.9999
Time = 0 vs. Time = 45	-9	No	ns	0.7142
Time = 15 vs. Time = 30	-5	No	ns	>0.9999
Time = 15 vs. Time = 45	-10	No	ns	0.4996
Time = 30 vs. Time = 45	-5	No	ns	>0.9999

Supplementary Table 3: Stability of DF_{GABAA} measurements made using ORCHID in astrocytes within a network state.

Network state: pre-SLE	
P value (paired t-test, two-tailed)	0.1756
Number of pairs (*n*)	10
Mean of differences ($-DF_{GABAA}$, mV)	-0.6490
SEM of differences ($-DF_{GABAA}$, mV)	0.4415
95% confidence interval ($-DF_{GABAA}$, mV)	-1.648 to 0.3497
Network state: post-SLE	
P value (paired t-test, two-tailed)	0.6190
Number of pairs (*n*)	8
Mean of differences ($-DF_{GABAA}$, mV)	-0.09516
SEM of differences ($-DF_{GABAA}$, mV)	0.1829
95% confidence interval ($-DF_{GABAA}$, mV)	-0.5278 to 0.3374
