# Supplementary material for: All-optical reporting of inhibitory receptor driving force in the nervous system
Source: Nat Commun. 2024 Oct 16;15:8913. doi: 10.1038/s41467-024-53074-y (PMC11484818; doi:10.1038/s41467-024-53074-y)
Supplement: Supplementary file 2 — Reporting Summary [file 41467_2024_53074_MOESM2_ESM.pdf]

nature portfolio

Corresponding author(s): Joseph Raimondo

Last updated by author(s): Aug 31, 2024

Reporting Summary

Nature Portfolio wishes to improve the reproducibility of the work that we publish. This form provides structure for consistency and transparency in reporting. For further information on Nature Portfolio policies, see our Editorial Policies and the Editorial Policy Checklist.

Statistics

For all statistical analyses, confirm that the following items are present in the figure legend, table legend, main text, or Methods section.

n/a	Confirmed	
[ ]	[x]	The exact sample size (*n*) for each experimental group/condition, given as a discrete number and unit of measurement
[ ]	[x]	A statement on whether measurements were taken from distinct samples or whether the same sample was measured repeatedly
[ ]	[x]	The statistical test(s) used AND whether they are one- or two-sided<br>*Only common tests should be described solely by name; describe more complex techniques in the Methods section.*
[x]	[ ]	A description of all covariates tested
[ ]	[x]	A description of any assumptions or corrections, such as tests of normality and adjustment for multiple comparisons
[ ]	[x]	A full description of the statistical parameters including central tendency (e.g. means) or other basic estimates (e.g. regression coefficient) AND variation (e.g. standard deviation) or associated estimates of uncertainty (e.g. confidence intervals)
[ ]	[x]	For null hypothesis testing, the test statistic (e.g. *F*, *t*, *r*) with confidence intervals, effect sizes, degrees of freedom and *P* value noted<br>*Give P values as exact values whenever suitable.*
[x]	[ ]	For Bayesian analysis, information on the choice of priors and Markov chain Monte Carlo settings
[x]	[ ]	For hierarchical and complex designs, identification of the appropriate level for tests and full reporting of outcomes
[x]	[ ]	Estimates of effect sizes (e.g. Cohen's *d*, Pearson's *r*), indicating how they were calculated

Our web collection on statistics for biologists contains articles on many of the points above.

Software and code

Policy information about availability of computer code

Data collection	Imaging data and patch clamp electrophysiology data were acquired using Micro-Manager version 2.0.0 (RRID:SCR_000415) and WinWCP version 5.6.6 (RRID:SCR_014713) respectively. Local-field potential recording data were acquired using LabChart 8 (RRID:SCR_017551).
Data analysis	Imaging and electrophysiology data analysis was performed using custom scripts written in MATLAB R2022b (RRID:SCR_001622). Statistical analysis for imaging and electrophysiology data was performed using GraphPad Prism version 7 (RRID:SCR_002798). Single nucleus RNA sequencing (snRNAseq) data was analyzed using Cell Ranger version 7.1.0 (RRID:SCR_017344) and the standard Seurat pipeline in R version 4.0.5 or 4.2.0 (RRID:SCR_001905). Code will be made publicly available on GitHub upon publication, and until then is available upon request.<br><br>Code for the simulations used Python version 3.12.1 (RRID:SCR_008394) and is accessible at: https://github.com/Eran707/Single-Cell-Simulator.

For manuscripts utilizing custom algorithms or software that are central to the research but not yet described in published literature, software must be made available to editors and reviewers. We strongly encourage code deposition in a community repository (e.g. GitHub). See the Nature Portfolio guidelines for submitting code & software for further information.

Data

Policy information about availability of data

All manuscripts must include a data availability statement. This statement should provide the following information, where applicable:

- Accession codes, unique identifiers, or web links for publicly available datasets
- A description of any restrictions on data availability
- For clinical datasets or third party data, please ensure that the statement adheres to our policy

Source data will be published alongside the publication of the manuscript and raw data is available upon reasonable request.

Research involving human participants, their data, or biological material

Policy information about studies with human participants or human data. See also policy information about sex, gender (identity/presentation), and sexual orientation and race, ethnicity and racism.

Reporting on sex and gender	No human participants, their data, or biological material were used in this study.
Reporting on race, ethnicity, or other socially relevant groupings	No human participants, their data, or biological material were used in this study.
Population characteristics	No human participants, their data, or biological material were used in this study.
Recruitment	No human participants, their data, or biological material were used in this study.
Ethics oversight	No human participants, their data, or biological material were used in this study.

Note that full information on the approval of the study protocol must also be provided in the manuscript.

Field-specific reporting

Please select the one below that is the best fit for your research. If you are not sure, read the appropriate sections before making your selection.

☒ Life sciences ☐ Behavioural & social sciences ☐ Ecological, evolutionary & environmental sciences

For a reference copy of the document with all sections, see nature.com/documents/nr-reporting-summary-flat.pdf

Life sciences study design

All studies must disclose on these points even when the disclosure is negative.

Sample size	No statistical method was used to predetermine sample sizes. Sample sizes were chosen based on accepted standards in the field (e.g., Alfonsa, H. et al. (2023) Intracellular chloride regulation mediates local sleep pressure in the cortex. Nat. Neurosci. 26, 64–78; Untiet, V. et al. (2023) Astrocytic chloride is brain state dependent and modulates inhibitory neurotransmission in mice. Nat. Commun. 14, 1871). We aimed to minimize the number of animals sacrificed while still being able to demonstrate the robustness of any effects, with sample size being sufficient to generate meaningful conclusions given biologically relevant effect sizes and typical data variance for the measures used.
Data exclusions	No acquired data were excluded, with three predetermined exceptions. For ORCHID recordings, cells which were visually determined to have blebbed during recordings were excluded. For in vitro data gathered using soma-directed puffs of GABA, recordings in which a movement artefact was present were excluded. Patch clamp recordings in which the access resistance was greater than 30 MOhm were excluded.
Replication	Several approaches were taken with regards to reproducibility. Where possible results were replicated with electrophysiology, with activation of endogenous GABA A receptors (GABAARs) on the cell soma, with theoretical modeling, with different cell types, and/or with gene expression data. A summary of replication measures taken follows:<br>1) During characterization of the ORCHID approach, imaging data were confirmed using patch clamp recordings. All attempts at replication were successful.<br>2) Endogenous inhibitory receptor driving force in different neuronal populations was measured using activation of endogenous GABAARs on the cell soma. These results were then replicated using ORCHID. All attempts at replication were successful.<br>3) A biophysical computational model of ion dynamics generated theoretical predictions regarding inhibitory receptor driving force, which were then replicated using activation of endogenous GABAARs, and using ORCHID. All attempts at replication were successful.<br>4) Dynamic inhibitory receptor driving forces were measured during seizure-like events, and the results in CaMKIIα+ pyramidal neurons were replicated in GAD2+ interneurons. All attempts at replication were successful.<br>5) Anion driving force in GFAP+ astrocytes was measured using ORCHID, and these results were confirmed through gene expression data using snRNAseq. SnRNAseq data consisted of four independent samples, each consisting of 36 hippocampal slices. All attempts at replication were successful.<br>6) In vivo determination of resting and dynamic inhibitory receptor driving forces were confirmed using local-field potential recordings. This data was acquired from a minimum of 4 to a maximum of 10 mice. All attempts at replication were successful.

Randomization	Mice (C57BL/6 background or GAD2-IRES-Cre) were randomly selected for hippocampal slices or imaging experiments, and were assigned randomly to experimental groups. Selection of cells for imaging was random, and within-cell comparisons were performed wherever possible. All experimental groups consisted of data from multiple hippocampal slices (≥ 4) or mice (≥ 4). This eliminated any slice- or animal-specific biases.
Blinding	Investigators were blinded to experimental conditions wherever it was possible during data acquisition and analysis. For voltage imaging and electrophysiology experiments, with the exception of the comparison of results in different cell types, all imaging data involve within-cell comparisons. It was not possible to completely blind the investigator to the cell type being investigated due to morphological differences, but the investigator was blinded to the promoter in use in each experimental group. All acquisition settings were identical for imaging experiments. Imaging and electrophysiology data analysis used a semi-automated pipeline in which the investigator was blinded to experimental group and conditions.

Reporting for specific materials, systems and methods

We require information from authors about some types of materials, experimental systems and methods used in many studies. Here, indicate whether each material, system or method listed is relevant to your study. If you are not sure if a list item applies to your research, read the appropriate section before selecting a response.

Materials & experimental systems

n/a	Involved in the study	
☐	☒	Antibodies
☒	☐	Eukaryotic cell lines
☒	☐	Palaeontology and archaeology
☐	☒	Animals and other organisms
☒	☐	Clinical data
☒	☐	Dual use research of concern
☒	☐	Plants

Methods

n/a	Involved in the study	
☒	☐	ChIP-seq
☒	☐	Flow cytometry
☒	☐	MRI-based neuroimaging

Antibodies

Antibodies used	*Describe all antibodies used in the study; as applicable, provide supplier name, catalog number, clone name, and lot number.*
Validation	*Describe the validation of each primary antibody for the species and application, noting any validation statements on the manufacturer's website, relevant citations, antibody profiles in online databases, or data provided in the manuscript.*

Animals and other research organisms

Policy information about studies involving animals; ARRIVE guidelines recommended for reporting animal research, and Sex and Gender in Research

Laboratory animals	The animals used in this study were wild-type (C57BL/6 background) or GAD2-IRES-Cre mice (RRID:IMSR_JAX:010802). Male and female mice were group housed on a 12 h/12 h light/dark cycle, with the lights on at 06:00. Food and water were provided ad libitum. Mouse pups were 6-8 days old when hippocampal slices were made. Adult mice were 4-8 weeks old at the time of experiments.
Wild animals	No wild animals were used in this study.
Reporting on sex	Mice of both sexes were used for all experiments. Sex was not recorded as a variable.
Field-collected samples	No field-collected samples were using in this study.
Ethics oversight	The use of animals was approved by the University of Cape Town Animal Ethics Committee (AEC Protocol 021/026 and AEC Protocol 022/038).

Note that full information on the approval of the study protocol must also be provided in the manuscript.

Plants

Seed stocks	No plants were used in this study.
Novel plant genotypes	No plants were used in this study.
Authentication	No plants were used in this study.
